# Japanese Patient Engagement Promotion Training (J‐PEPT): Learning course on the implementation strategy of patient engagement

**DOI:** 10.1002/jgf2.665

**Published:** 2024-02-12

**Authors:** Masaru Kurihara, Shintaro Kosaka, Yusuke Yasumoto, Akie Yamaguchi, Tomomi Yoshida, Ayako Iyasu, Hideyuki Kashiwagi, Toru Kimura, Kiichi Enomoto, Kiyomi Tanno, Keiko Inoue, Yaeko Ishihara, Noriko Iwaya, Aoki Takuya

**Affiliations:** ^1^ Department of Patient Safety Nagoya University Hospital Aichi Japan; ^2^ Department of Hospital Medicine Hiroo Hospital Tokyo Japan; ^3^ Department of General Medicine Itabashi Chuo Medical Center Tokyo Japan; ^4^ Department of Pharmacy Tokachi‐kin‐ikyo Obihiro Hospital Obihiro Japan; ^5^ University of Tsukuba Graduate School Systems and Information Engineering Ibaraki Japan; ^6^ Nagi Elementary School Okayama Japan; ^7^ Department of Transitional and Palliative Care Iizuka hospital Fukuoka Japan; ^8^ Department of Rehabilitation Nerima Hikarigaoka Hospital Tokyo Japan; ^9^ Department of Pharmacy Nerima Hikarigaoka Hospital Tokyo Japan; ^10^ Division of Supportive Care, Survivorship Group National Cancer Center Institute for Cancer Control Tokyo Japan; ^11^ Association for Medical Malpractice Victims Tokyo Japan; ^12^ Fabry NEXT Aichi Japan; ^13^ Intractable Disease Society Support Familia Yamaguchi Yamaguchi Japan; ^14^ Division of Clinical Epidemiology, Research Center for Medical Sciences The Jikei University School of Medicine Tokyo Japan

## Abstract

Patient engagement for patient safety is emphasized in recent years. Therefore, the Committee on Quality and Patient Safety of the Japan Primary Care Association developed a Japanese Patient Engagement Promotion Training (J‐PEPT) course. J‐PEPT promotes to facilitate the implementation of PE strategies and contributes to nationwide dissemination for patient safety.

To the Editor,

Since the end of the 20th century, patient safety has become a global issue.^1^ Although many patient safety measures (such as changing the behavior of healthcare providers) have been taken, the role of patients in safety measures has also become more important in recent years. Patient engagement (PE) is defined as patients, families, their representatives, and health professionals working in active partnerships at various levels across the healthcare system to improve health and healthcare.^2^ PE to address safety issues that cannot be resolved by healthcare providers alone is considered by policy makers and healthcare providers. However, in Japan, there are barriers to improving patient safety, and one of them is the emphasis on patient centeredness, including PE.^1^ Translating patient‐centered safety measures including PE from theory to implementation is challenging, making it a global issue; thus, it is important to create learning courses where PE can be taught. Therefore, to promote the PE implementation strategies in primary care, which have traditionally emphasized patient centeredness, we report a Japanese Patient Engagement Promotion Training (J‐PEPT) course developed by the Committee on Quality and Patient Safety of the Japan Primary Care Association.

J‐PEPT aims to provide participants with the necessary knowledge and skills to implement PE strategies in healthcare settings. It is held about twice a year and has been attended by more than 60 people from across the country to date. Participants include physicians, nurses, pharmacists, as well as patients. PE has been also emphasized when planning, involving not only healthcare professionals (including physicians), but also patients, families, and social scientists from various fields. This is designed such that participants can learn while incorporating diverse opinions, deepening their understanding of the importance of PE.

J‐PEPT covers many topics regarding PE, including sessions on implementing PE in primary care using the “guide to improving patient safety in primary care settings by engaging patients and families”,^3^ utilizing patient experiences, and exploring PE in telemedicine. Several measures have been taken to enhance J‐PEPT's implementation in healthcare settings: first, interactive opportunities are provided to offer participants a chance to learn how to promote implementation in their respective healthcare settings. Second, a mindset that can serve as an implementation champion within healthcare institutions is conveyed. Finally, opportunities for patient input are always provided to participants to spark insights, treating the course itself as an opportunity for PE.

In each session, learning objectives are set, and a survey using a 5‐point Likert scale (1: “strongly disagree” to 5: “strongly agree”) is conducted to assess whether these objectives have been achieved. In May 2023, the average score of 4.2 was evaluated, with 17 of 18 participants (94.4%) rating it as four or five. Additionally, 15 of 18 participants (83.3%) indicated that they understood PE. These respondents included doctors, pharmacists, and patients.

While PE offers various benefits, such as improving health outcomes, enhancing patient care, and reducing costs,^4^ its implementation and opportunity to learn have been limited. In Japan, tools such as the medication notebook can be utilized for PE. Therefore, the effective use of existing resources on J‐PEPT is expected. As PE is highlighted as the theme for World Patient Safety Day 2023,^5^ and with the growing global momentum, J‐PEPT promotes to facilitate the implementation of PE strategies and contributes to nationwide dissemination for patient safety.

## CONFLICT OF INTEREST STATEMENT

None.

## References

[1] Kurihara M , Watari T , Kosaka S , Enomoto K , Kimura T , Taniguchi K , et al. Root cause analysis to identify major barriers to the promotion of patient safety in Japan. J Patient Saf Risk Manag. 2023;28(1):9–14.

[2] Carman KL , Dardess P , Maurer M , Sofaer S , Adams K , Bechtel C , et al. Patient and family engagement: a framework for understanding the elements and developing interventions and policies. Health Aff. 2013;32(2):223–231. 10.1377/hlthaff.2012.1133 23381514

[3] Smith K , Baker K , Wesley D , Zipperer L , Clark MD , Hanneke CR , et al. Guide to Improving Patient Safety in Primary Care Settings by Engaging Patients and Families. Agency for Healthcare Research and Quality Published online. 2017.

[4] Marzban S , Najafi M , Agolli A , Ashrafi E . Impact of patient engagement on healthcare quality: a scoping review. J Patient Exp. 2022;9:23743735221125439. 36134145 10.1177/23743735221125439 PMC9483965

[5] World Patient Safety Day 2023: Engaging Patients for Patient Safety. https://www.who.int/news‐room/events/detail/2023/09/17/default‐calendar/world‐patient‐safety‐day‐2023‐‐engaging‐patients‐for‐patient‐safety

